# Antitumor effect of the novel sphingosine kinase 2 inhibitor ABC294640 is enhanced by inhibition of autophagy and by sorafenib in human cholangiocarcinoma cells

**DOI:** 10.18632/oncotarget.7914

**Published:** 2016-03-04

**Authors:** Xiwei Ding, Roongruedee Chaiteerakij, Catherine D. Moser, Hassan Shaleh, Jeffrey Boakye, Gang Chen, Albert Ndzengue, Ying Li, Yanling Zhou, Shengbing Huang, Frank A. Sinicrope, Xiaoping Zou, Melanie B. Thomas, Charles D. Smith, Lewis R. Roberts

**Affiliations:** ^1^ Department of Gastroenterology, The Affiliated Drum Tower Hospital of Nanjing University Medical School, Nanjing, China; ^2^ Division of Gastroenterology and Hepatology, College of Medicine, Mayo Clinic and Mayo Clinic Cancer Center, Rochester, MN, USA; ^3^ Department of Medicine, Faculty of Medicine, Chulalongkorn University and King Chulalongkorn Memorial Hospital, Thai Red Cross Society, Bangkok, Thailand; ^4^ Hollings Cancer Center, Division of Hematology-Oncology, Department of Medicine, Medical University of South Carolina, Charleston, SC, USA; ^5^ Apogee Biotechnology Corporation, Hummelstown, PA, USA

**Keywords:** cholangiocarcinoma, sphingosine kinase 2, ABC294640, autophagy, sorafenib

## Abstract

Sphingosine kinase 2 (Sphk2) has an oncogenic role in cancer. A recently developed first-in-class Sphk2 specific inhibitor ABC294640 displays antitumor activity in many cancer models. However, the role of Sphk2 and the antitumor activity of its inhibitor ABC294640 are not known in cholangiocarcinoma. We investigated the potential of targeting Sphk2 for the treatment of cholangiocarcinoma. We found that Sphk2 is overexpressed in five established human cholangiocarcinoma cell lines (WITT, HuCCT1, EGI-1, OZ and HuH28) and a new patient-derived cholangiocarcinoma cell line (LIV27) compared to H69 normal cholangiocytes. Inhibition of Sphk2 by ABC294640 inhibited proliferation and induced caspase-dependent apoptosis. Furthermore, we found that ABC294640 inhibited STAT3 phosphorylation, one of the key signaling pathways regulating cholangiocarcinoma cell proliferation and survival. ABC294640 also induced autophagy. Inhibition of autophagy by bafilomycin A1 or chloroquine potentiated ABC294640-induced cytotoxicity and apoptosis. In addition, ABC294640 in combination with sorafenib synergistically inhibited cell proliferation of cholangiocarcinoma cells. Strong decreases in STAT3 phosphorylation were observed in WITT and HuCCT1 cells exposed to the ABC294640 and sorafenib combination. These findings provide novel evidence that Sphk2 may be a rational therapeutic target in cholangiocarcinoma. Combinations of ABC294640 with sorafenib and/or autophagy inhibitors may provide novel strategies for the treatment of cholangiocarcinoma.

## INTRODUCTION

Cholangiocarcinoma (CCA) is a highly malignant adenocarcinoma with features of cholangiocyte differentiation. The incidence of this neoplasm appears to be increasing in several Western countries [1–3]. Over the past 3 decades, 5-year survival rates of CCA have not increased and remain at 10% [4]. Although surgical resection and liver transplantation are potentially curative therapies for selected patients, most patients are diagnosed at late stages and are not eligible for these options. In addition, conventional chemotherapy and radiotherapy are suboptimal in prolonging long-term survival of CCA patients. Thus, there is an urgent need to develop novel effective therapeutic strategies against this neoplasm.

Sphingosine kinases (Sphk) are lipid kinases that regulate the sphingolipid metabolic pathway and have been shown to play an oncogenic role in numerous cancers [5–9]. The Sphk isoforms (Sphk1 and Sphk2) catalyze the conversion of the pro-apoptotic sphingolipid sphingosine to the mitogenic lipid sphingosine-1-phosphate (S1P). S1P has been proposed to contribute to cancer development and progression by regulating tumor proliferation, migration and angiogenesis [10]. Sphk therefore represents a potential novel target for cancer therapeutics. Recent studies suggest that S1P, probably generated from Sphk2, may promote CCA proliferation and migration, suggesting Sphk2 and S1P as potential therapeutic targets in CCA [11].

ABC294640 is a novel Sphk2 specific inhibitor with high efficacy in several preclinical models of cancer and synergistic anticancer activity with chemotherapies or molecular targeted therapies [9, 12–20]. It has excellent oral bioavailability and biodistribution *in vivo* and shows promising results with feasible tolerance in Phase I clinical trial. Of note, one metastatic cholangiocarcinoma patient receiving ABC294640 had stabilization of disease for 16 months. Additionally, ABC294640 is highly selective for the Sphk2 isoform at concentrations up to at least 100 μM [15].

Autophagy is a conserved catabolic degradation process whereby cellular organelles and proteins are engulfed by autophagosomes, digested in lysosomes, and recycled to maintain cellular metabolism. Sustained autophagy may result in cell death. ABC294640 has been shown to induce cancer cell death by both apoptotic and autophagic pathways [17, 21]. However, emerging evidence suggests that autophagy can also enable cell survival and lead to treatment resistance [22–24].

Sorafenib is a FDA-approved multikinase inhibitor for the treatment of hepatocellular carcinoma and renal cell carcinoma. Studies suggest that sorafenib also has a tumor suppression role in CCA in part through inhibition of STAT3 signaling pathway [25, 26]. ABC294640 has been shown to have an additive to synergistic effect with sorafenib in inhibiting tumor growth in hepatocellular carcinoma and pancreatic adenocarcinoma cells [13, 14].

Therefore, we decided to investigate the following: (1) whether pharmacological inhibition of Sphk2 by ABC294640 inhibits CCA cell growth; (2) whether ABC294640 modulates apoptosis and autophagy in CCA cells; (3) whether induction of autophagy in CCA cells has a pro-survival or pro-death effect; (4) whether ABC294640 has a synergistic effect with sorafenib in CCA cells.

## RESULTS

### Sphk2 is overexpressed in cholangiocarcinoma cells

**Figure 1:**
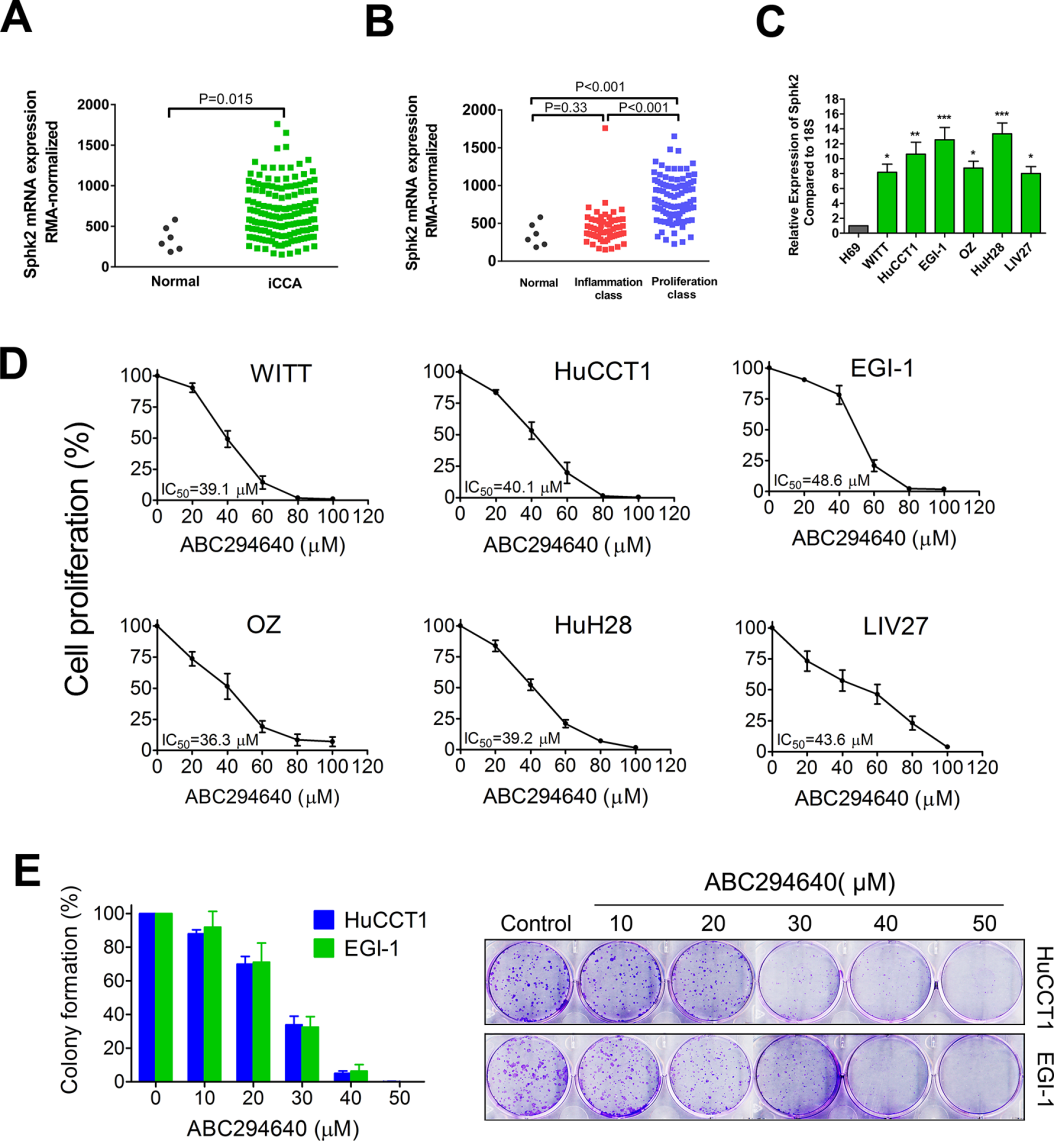
Sphk2 is overexpressed in cholangiocarcinoma cells and promotes cell proliferation (**A**) Publicly available microarray dataset GSE32225 was downloaded and the Sphk2 expression levels between human normal biliary epithelial cells (*n* = 6) or intrahepatic cholangiocarcinoma (iCCA) (*n* = 149) were compared. (**B**) Sphk2 expression level of normal human biliary epithelial cells (*n* = 6) or two subclasses of iCCA (inflammation class, *n* = 57; proliferation class, *n* = 92) were compared. (**C**) mRNA expression of Sphk2 in human cholangiocarcinoma cell lines and the H69 normal human cholangiocyte cell line were analyzed by real-time PCR. 18S was used as the internal control.*P < 0.05; **P < 0.01; ***P < 0.001, compared with H69 cells. (**D**) Cells were treated with ABC294640 at different concentrations (20–100 μM) for 72 h and cell proliferation was determined by BrdU ELISA assay. (**E**) Plated HuCCT1 and EGI-1 cells were treated with ABC294640 at different concentrations (10–50 μM) for 7 days and colonies were stained with 0.5% crystal violet. The results are presented as mean ± SEM from at least three independent experiments.

To determine the potential utility of targeting Sphk2 for the treatment of CCA, we measured the gene expression levels of Sphk2 in CCA cells. We first analyzed Sphk2 mRNA expression in a publicly available CCA microarray data set GSE32225 originated by the University of Barcelona. This dataset contained microarray mRNA gene profiles on human normal biliary epithelial cells (*n* = 6) or intrahepatic CCA (iCCA) (*n* = 149). Robust Multi-array Average (RMA)-normalized gene expression data were used to compare the Sphk2 expression level between normal subjects and iCCA patients. As shown in Figure 1A, Sphk2 expression was increased in iCCA patients compared to normal subjects (*P* = 0.015). Through an integrative genomic analysis, Sia D et al. identified 2 classes of intrahepatic CCA, the proliferation class and the inflammation class. The proliferation class was accociated with a worse survival. We found that Sphk2 mRNA expression was mainly elevated in the proliferation class (Figure 1B), suggesting that high Sphk2 mRNA expression may be associated with worse survival. In addition, we measured the mRNA expression levels of Sphk2 in both established human CCA lines (WITT, HuCCT1, EGI-1, OZ and HuH28) and one new patient-derived CCA cell line (LIV27), as well as in a normal human cholangiocyte cell line (H69). As shown in Figure 1C, all CCA cell lines expressed high levels of Sphk2 mRNA compared to H69 cells. The expression of Sphk2 was 8 to 13-fold increased in CCA cell lines compared to H69 cells. These data demonstrate that Sphk2 is overexpressed in CCA cells.

### ABC294640 inhibits cell proliferation and clonogenicity of cholangiocarcinoma cells

To assess the effect of Sphk2 inhibition on CCA cell growth, CCA cells were exposed to increasing concentrations of ABC294640 for 72 h and cell proliferation was evaluated by BrdU ELISA assay. All cell lines tested showed a potent dose-dependent growth inhibition after ABC294640 treatment (Figure 1D). The IC_50_ values of ABC294640 ranged from 36.3 μM to 48.6 μM. The HuCCT1 and EGI-1 cells have the capability to form colonies when plated. To test the effect of ABC294640 on clonogenicity, HuCCT1 and EGI-1 cells were treated with increasing concentrations of ABC294640 for 7 days, stained with crystal violet and colonies were counted. ABC294640 was more potent in the clonogenicity assay (Figure 1E). Additionally, RNA interference was used to confirm the role of Sphk2 in CCA cell proliferation. Treatment of HuCCT1 cells with siRNA specific for Sphk2 effectively suppressed the expression of the target mRNA and inhibited cell growth (Supplementary Figure 1).

### ABC294640 induces apoptosis of cholangiocarcinoma cells

**Figure 2:**
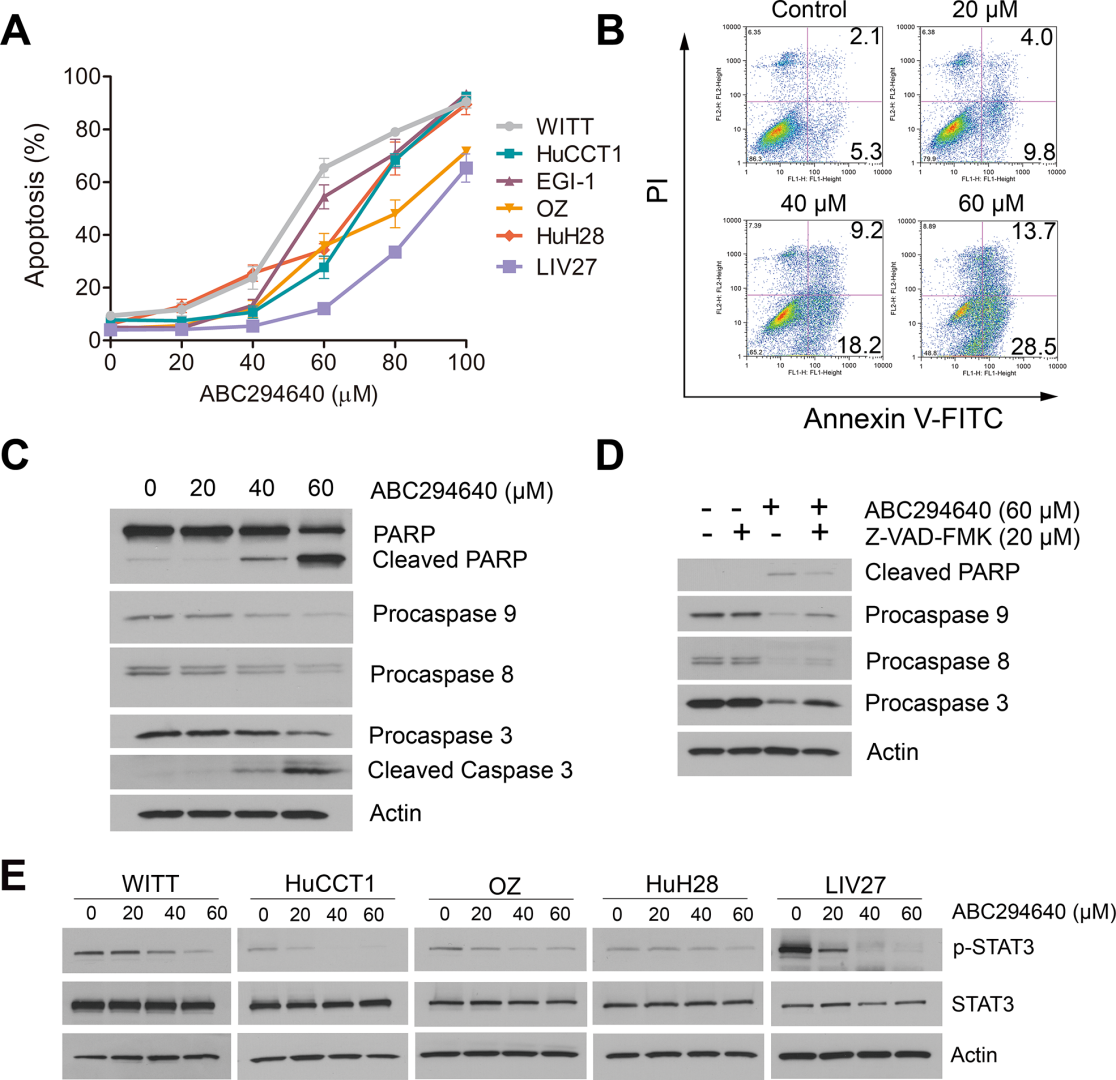
ABC294640 induces apoptosis and inhibits STAT3 phosphorylation in cholangiocarcinoma cells (**A**) Percentage apoptosis of cholangiocarcinoma cells treated with the indicated concentrations of ABC294640 (20–100 μM) for 72 h. Apoptosis was quantified by assessing characteristic nuclear changes of apoptosis by fluorescence microscopy after DAPI staining. The results are presented as mean ± SEM from at least three independent experiments. (**B**) WITT cells were treated with 20-60 μM ABC294640 for 72 h. Apoptosis was then measured by Annexin V-FITC/PI labeling followed by flow cytometry. (**C**) Western immunoblots for PARP and caspases 9, 8 and 3 performed on cell lysates from the same treatment as the flow cytometry assay. (**D**) Western immunoblots for PARP and caspases 9, 8 and 3 performed on WITT cell lysates prepared after 72 h treatment with ABC294640 in the presence or absence of the pan-caspase inhibitor, Z-VAD-FMK. (**E**) Cholangiocarcinoma cells were treated with 20–60 μM ABC294640 for 48 h. STAT3 Y705 phosphorylation was determined by immunoblotting.

To further characterize ABC294640-induced cytotoxicity, apoptotic cell death was assessed by DAPI staining. As shown in Figure 2A, ABC294640 dose-dependently induced apoptosis of all CCA cell lines after 72 h treatment. The induction of apoptosis was validated by Annexin V/PI double staining in WITT cells (Figure 2B). Increase in apoptosis of WITT cells by treatment with ABC294640 was further confirmed by the dose-dependent increase of the cleavage of caspase3 and PARP (Figure 2C). Furthermore, pretreatment of cells with the pan-caspase inhibitor, Z-VAD-FMK, partly blocked ABC294640-induced apoptosis (Figure 2D), indicating ABC294640-induced apoptosis partially depends on caspase activation.

### ABC294640 inhibits STAT3 signaling

The STAT3 pathway is a signaling pathway regulating cell proliferation and apoptosis in CCA cells and has been shown to be an important mediator of tumor resistance [27–29]. Previous studies have shown that Tyr705-phosphorylation of STAT3 is required for its nuclear accumulation, and consequently its transcriptional activation of anti-apoptotic proteins. We examined the levels of phosphorylated STAT3 after ABC294640 treatment by immunoblotting (Figure 2E). Our results showed that ABC294640 downregulated STAT3 phosphorylation in a dose-dependent manner.

### ABC294640 induces autophagy in cholangiocarcinoma cells

**Figure 3:**
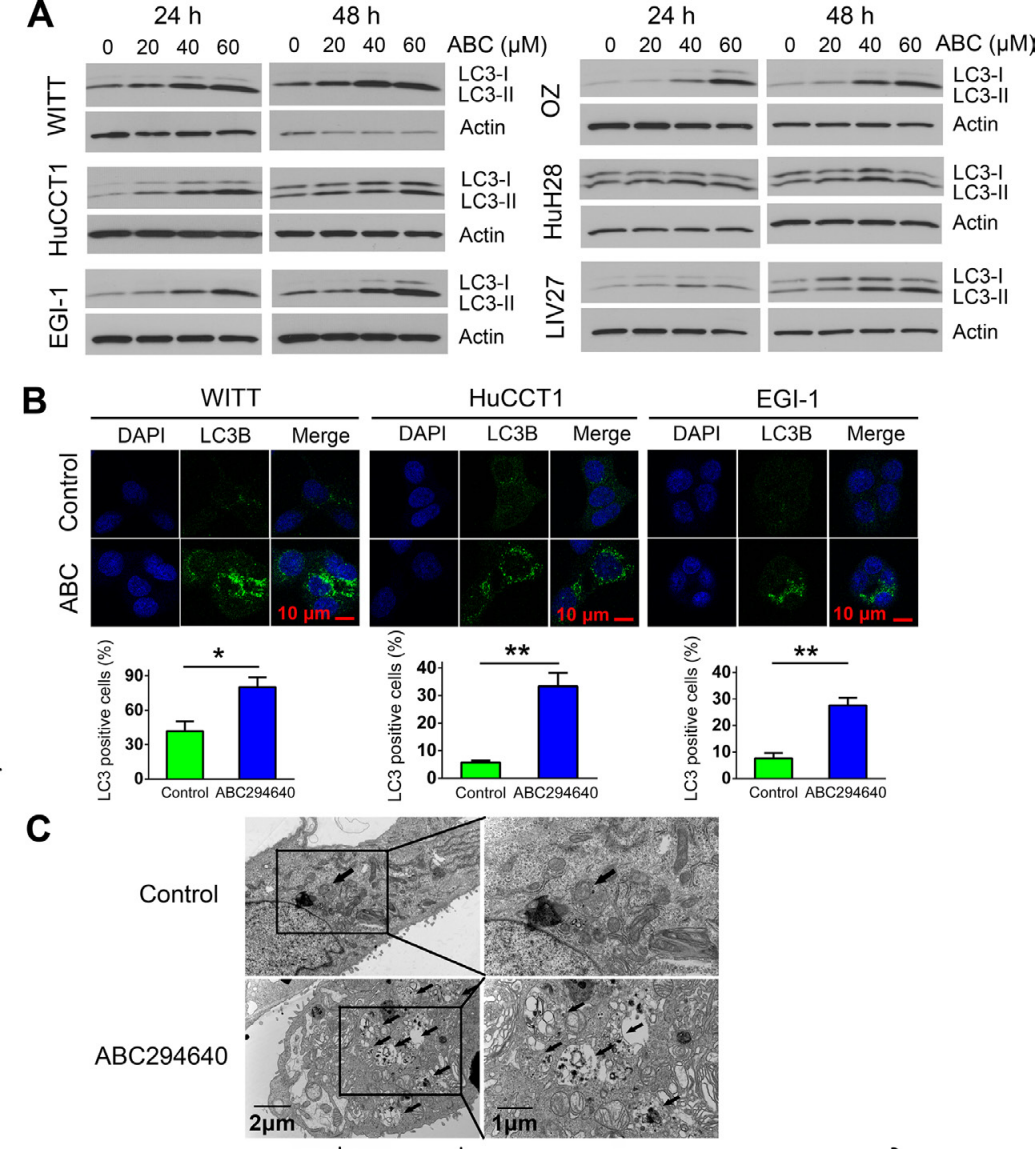
ABC294640 induces autophagy in cholangiocarcinoma cells (**A**) WITT, HuCCT1, EGI-1, OZ, HuH28 and LIV27 cells were treated with 20–60 μM ABC294640 for 24 h and 48 h. Autophagy was assessed by the conversion of cytosolic LC3-I into membrane-bound LC3-II by immunoblotting. (**B**) WITT, HuCCT1 and EGI-1 cells were treated with 40 μM ABC294640 for 24 h. LC3 detected by immunostaining was analyzed and compared with controls. Quantification of puncta is shown (lower panel). The results are presented as mean ± SEM from at least three independent experiments.*P < 0.05; **P < 0.01; ***P < 0.001. (**C**) EGI-1 cells were treated with 60 μM ABC294640 for 24 h and subjected to transmission electron microscopy. Representative micrographs at low magnification (left) or high magnification (right) are shown. The arrows show autophagic vacuoles.

**Figure 4:**
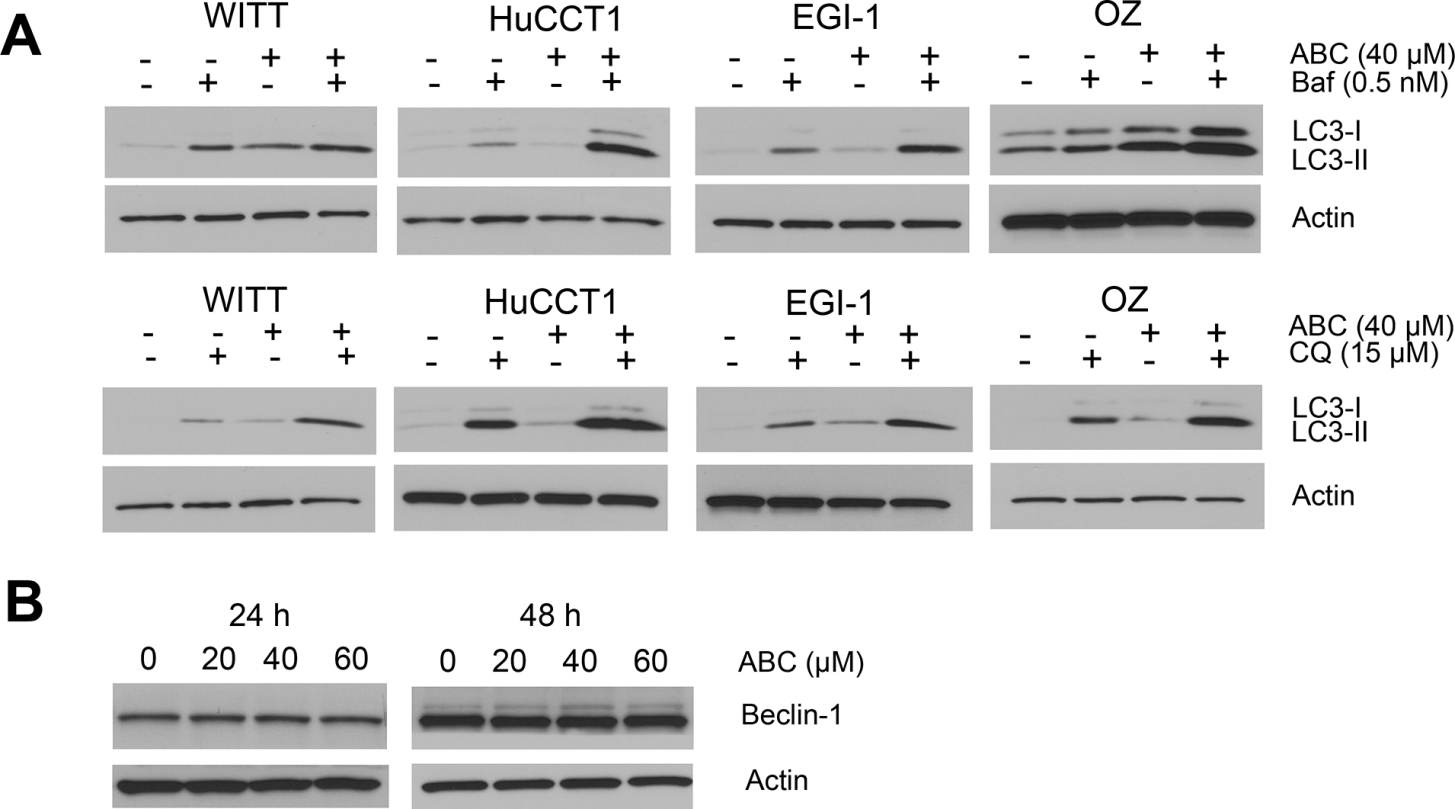
ABC294640 activates autophagic flux in cholangiocarcinoma cells (**A**) Cells were treated with 40 μM ABC294640 (ABC) alone or in combination with 0.5 nM bafilomycin A1 (Baf) or 15 μM chloroquine (CQ) for 48 h and cell lysates were subjected to immunoblotting. Autophagic flux results in substantial accumulation of LC3-II. (**B**) OZ cells were treated with ABC294640 (20–60 μM) for 24 h and 48 h. Beclin-1 expression was measured by immunoblotting.

Autophagy is a lysosome-mediated degradation process important for maintaining cellular homeostasis. It has been reported that cancer cells undergo autophagy in response to various anticancer treatments. ABC294640 has previously been shown to induce autophagic cell death in cancer cells [21]. Thus, we examined whether ABC294640 induces autophagy in CCA cells. First, we examined the expression of autophagosome marker LC3-II using immunoblotting. Expression of LC3-II increased in all CCA cells treated with ABC294640 in a dose-dependent manner at both 24 and 48 h (Figure 3A). The autophagosome-specific protein LC3 can also be detected by immunofluorescence. When autophagy is induced, LC3-II distributes to the membrane of autophagosomes and shows a characteristic punctate staining pattern. We therefore treated WITT, HuCCT1 and EGI-1 cells with 40 μM ABC294640 for 24 h and determined the localization of LC3 using fluorescence microscopy. In untreated tumor cells, LC3 showed a diffuse staining pattern, whereas cells treated with ABC294640 demonstrated a punctate staining pattern consistent with autophagy. Quantitative analysis showed that the percentage of cells with LC3 dots increased significantly in WITT, HuCCT1 and EGI-1 cell lines after treatment with ABC294640 (Figure 3B). In addition, electron microscopy analysis showed that autophagic vacuoles containing cytoplasmic materials increased in EGI-1 cells treated with 60 μM ABC294640 for 24 h compared with untreated control cells (Figure 3C). Moreover, addition of the lysosome inhibitor Bafilomycin A1 (Baf) or chloroquine (CQ) resulted in further accumulation of LC3-II in these cell lines as compared to cells treated with single agent, indicating of autophagic flux (Figure 4A). Together, these results indicate that ABC294640 induces autophagy in CCA cells. We also measured the levels of Beclin-1 (another key protein in autophagy pathway) in OZ cells exposed to ABC294640 by immunoblotting. Although ABC294640 increased Beclin-1 expression in kidney carcinoma cells [21], it did not affect Beclin-1 expression in CCA cells (Figure 4B). Therefore, autophagy in CCA cells induced by ABC294640 does not result from changes in the Beclin-1 expression level.

### Inhibition of autophagy enhances the anticancer effect of ABC294640 in cholangiocarcinoma cells

**Figure 5:**
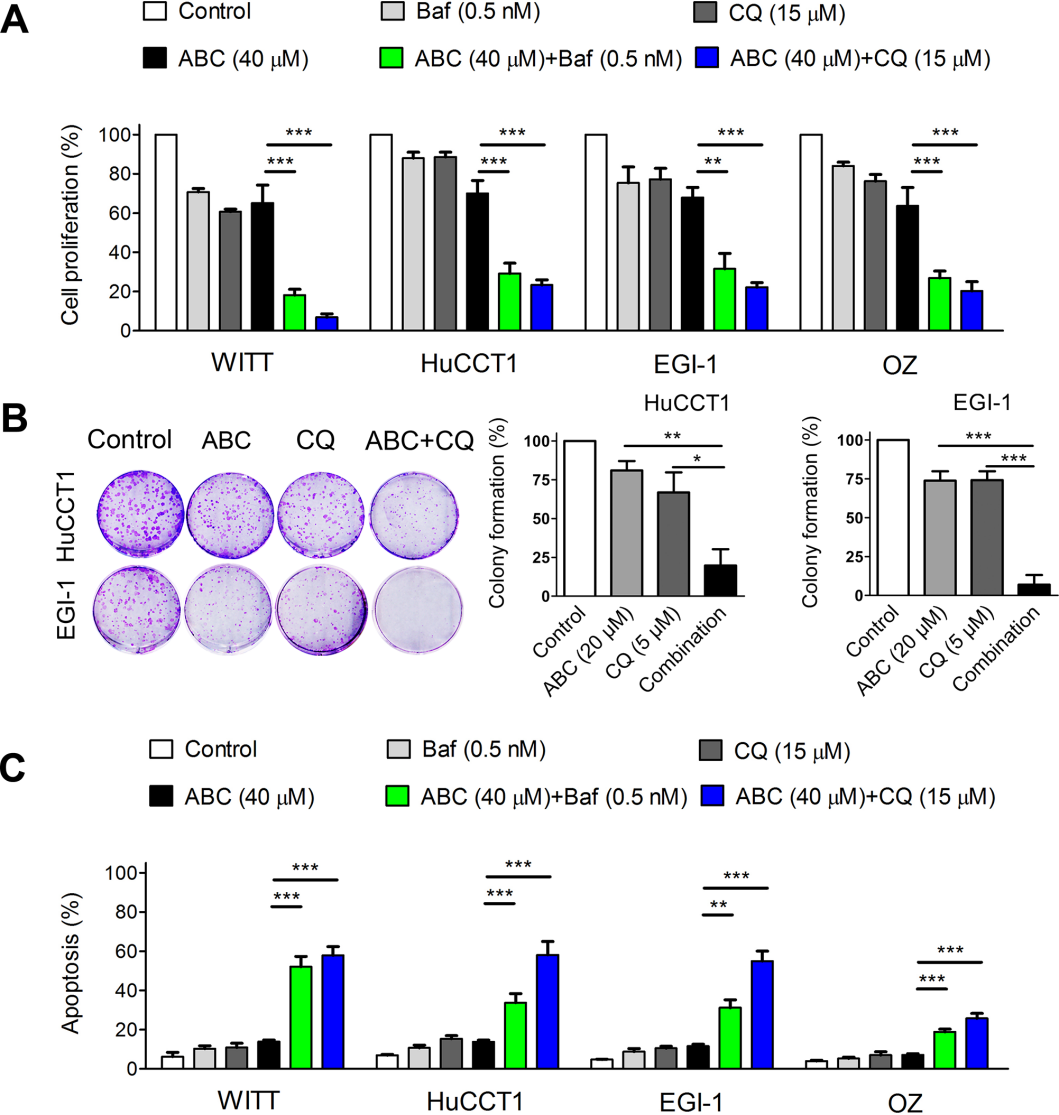
Targeting autophagy potentiates ABC294640-induced cytotoxicity and apoptosis in cholangiocarcinoma cells (**A**) WITT, HuCCT1, EGI-1 and OZ cells were treated with 40 μM ABC294640 (ABC) alone or in combination with 0.5 nM bafilomycin A1 (Baf), or 15 μM chloroquine (CQ) for 48 h. Cell proliferation was then determined by the BrdU ELISA assay. (**B**) HuCCT1 and EGI-1 cells were treated with 20 μM ABC294640 alone or in combination with 5 μM CQ for 7 days. Cell proliferation was analyzed using the colony formation assay. (**C**) WITT, HuCCT1, EGI-1 and OZ cells were treated with 40 μM ABC294640 alone or in combination with 0.5 nM Baf, or 15 μM CQ for 48 h. Cell apoptosis was quantified by DAPI staining. The results are presented as mean ± SEM from at least three independent experiments. *P < 0.05; **P < 0.01; ***P < 0.001.

Autophagy can result in diverse outcomes depending on the context and stimulus. It can function as a caspase-independent cell death mechanism. In contrast, it can also protect cancer cells from induction of cell death by various therapies. To determine the role of autophagy in cholangiocarcinoma cell survival during ABC294640 treatment, we evaluated the combined effects of the autophagy inhibitors Baf or CQ and ABC294640. The effects of ABC294640 with and without autophagy inhibition on cell proliferation were assessed by BrdU ELISA assay. As shown in Figure 5A, combined treatment with autophagy inhibitors and ABC294640 further decreased cell proliferation of WITT, HuCCT1, EGI-1 and OZ cells. We also performed colony formation assays in which HuCCT1 and EGI-1 cells were treated with low concentrations of ABC294640 (20 μM) and CQ (5 μM), either alone or in combination. In HuCCT1 cells, there was a 19% decrease in colony formation after ABC294640 (20 μM) treatment and a 33% decrease in colony formation after CQ (5 μM) treatment, as compared with untreated cells. Further, the combination of ABC294640 (20 μM) and CQ (5 μM) induced an 80% decrease in colony formation compared with no treatment (Figure 5B). Similar results were observed in EGI-1 cells treated with ABC294640 (20 μM) and CQ (5 μM). To determine whether autophagy inhibition triggers apoptosis, we assessed apoptosis by DAPI staining in cells treated with ABC294640 or Baf and CQ, either alone or in combination. Autophagy inhibitors significantly enhanced ABC294640-induced apoptosis (Figure 5C). Of note, the concentration of bafilomycin A1 we used was extremely low which was less likely to have off-target effects. Altogether, these results suggest that autophagy functions as a survival mechanism during ABC294640 treatment and that inhibition of autophagy enhances the anticancer effect of ABC294640 in CCA cells.

### ABC294640 has a synergistic effect with sorafenib

**Figure 6:**
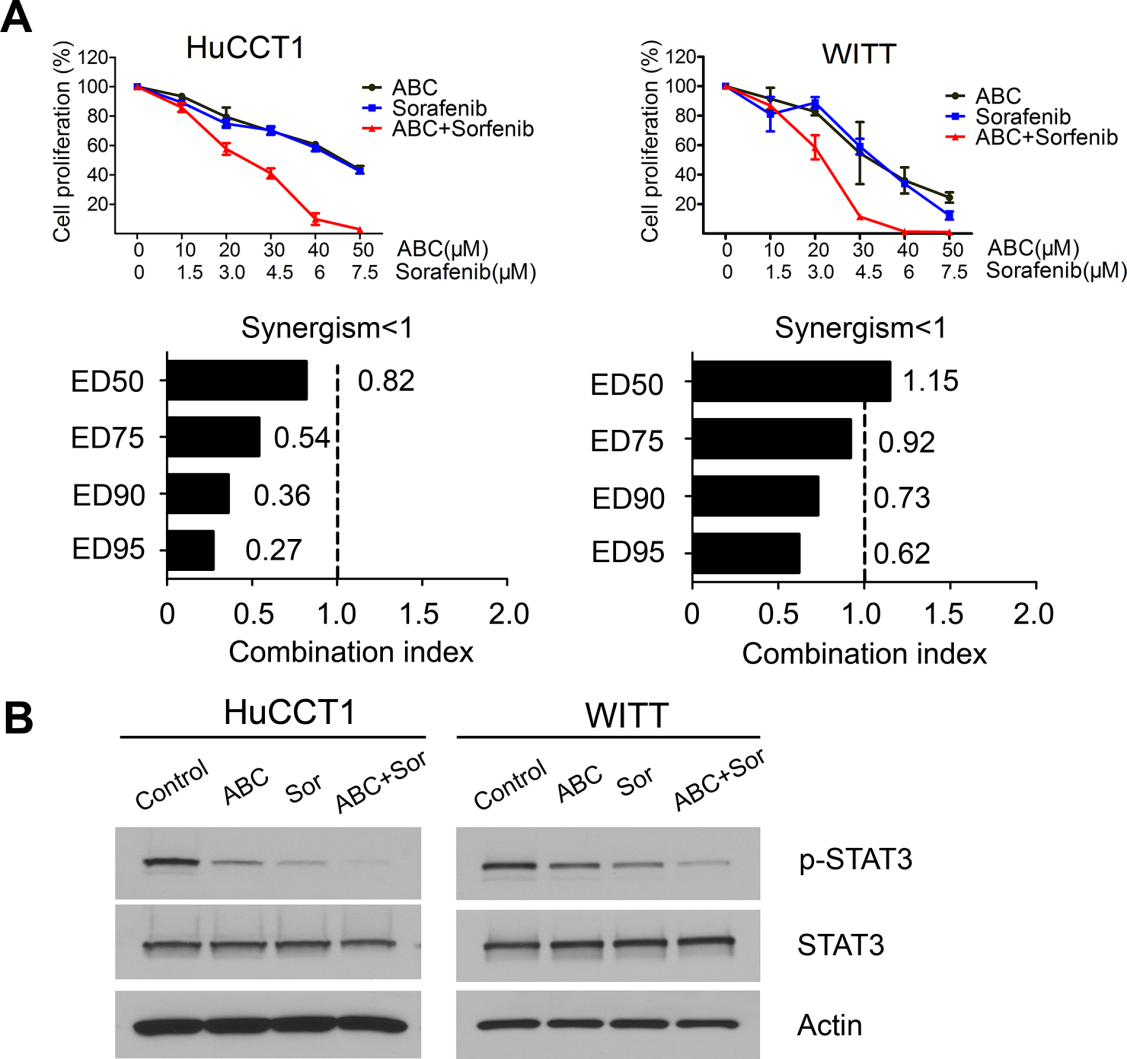
ABC294640 in combination with sorafenib synergistically inhibits proliferation in cholangiocarcinoma cells (**A**) HuCCT1 and WITT cells were treated with different fixed-ratio combinations of ABC294640 and sorafenib and proliferation was determined by BrdU ELISA assay. The results are presented as mean ± SEM from at least three independent experiments. The combination index (CI) was determined using the Chou-Talalay Method. CI < 1 indicates that the interaction between ABC294640 and sorafenib was synergistic. (**B**) HuCCT1 and WITT cells were treated with 40 μM ABC294640 (ABC) and/or 6 μM sorafenib (Sor) for 24 h and whole cell lysates were then subjected to immunoblotting.

**Figure 7:**
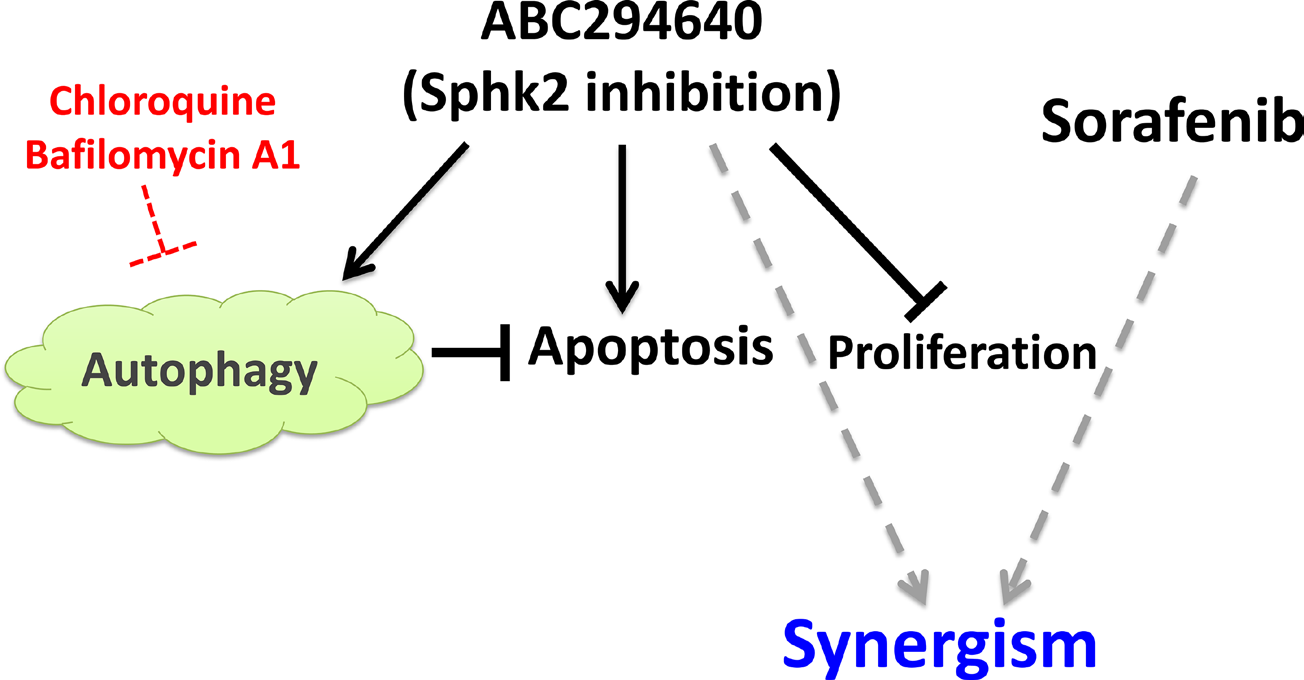
Schematic representation of the effect of Sphk2 specific inhibitor ABC294640 on CCA ABC294640 inhibits proliferation, induces apoptosis and autophagy in CCA cells. Autophagy inhibitors potentiates ABC294640-induced cytotoxicity and apoptosis. ABC294640 has a synergistic effect with sorafenib in inhibiting cell proliferation.

Because both ABC294640 and sorafenib decrease CCA cell growth by interfering with the pro-survival STAT3 pathway, we hypothesized that the combination of the two drugs may result in synergistic cytotoxic effects. Therefore, HuCCT1 and WITT cell lines were treated with increasing concentrations of ABC294640 in a fixed-ratio with sorafenib. Following 72 h of treatment, cell proliferation was assessed and the CI was calculated to determine whether the drug combination resulted in synergistic toxicity. In HuCCT1 cells, the combination of ABC294640 and sorafenib resulted in moderate to strong synergism (Figure 6A). In WITT cells, the combination of ABC294640 with sorafenib resulted in moderate synergism, particularly at higher concentrations (Figure 6A). Therefore, ABC294640 synergistically increases the cytotoxicity of sorafenib in cholangiocarcinoma cells. To gain insight into the mechanisms underlying the combined cytotoxicity of these agents, the effects of ABC294640 and sorafenib on the STAT3 phosphorylation were examined by immunoblotting. Treatment with the combination of ABC294640 and sorafenib resulted in more profound decreases in STAT3 phosphorylation in HuCCT1 and WITT cells than treatment with either agent alone (Figure 6B). These findings indicate that combined treatment of CCA cells with ABC294640 and sorafenib leads to more effective suppression of pro-survival STAT3 signaling and inhibition of cancer cell growth than either agent alone. The effects of the combinations of ABC294640 with autophagy inhibitors and sorafenib are illustrated in Figure 7.

## DISCUSSION

The oncogenic role of Sphk1 has been explored in a range of cancers [6, 30–32]. In contrast, the role of Sphk2 in cancer has not been well characterized. Despite several initial studies reported pro-apoptotic effects of Sphk2 [33], recent evidence are emerging that implicate Sphk2 in the development of many tumors. Inhibition of Sphk2 is observed to suppress colitis-driven colon cancer in mice [34]. Sphk2-deficient MCF-7 breast cancer cells grow poorly *in vivo* [35]. Sphk2 has also been shown to have an oncogenic role in acute lymphoblastic leukemia by enhancing MYC expression [9]. Furthermore, the Sphk2 specific inhibitor ABC294640 displays anticancer effects in a number of cellular and animal models and is currently in phase II clinical trials for the treatment of advanced solid tumors. Therefore, targeting Sphk2 appears to have therapeutic potential for treating cancer.

In this study, we first found that Sphk2 is over expressed in CCA cells, suggesting that Sphk2 may have an oncogenic role in this neoplasm. We therefore investigated the potential effect of targeting Sphk2 by its novel specific inhibitor ABC294640 in CCA cells. We found that ABC294640 suppresses proliferation of six CCA cell lines. Knock down of Sphk2 using specific siRNA confirmed the role of Sphk2 in the growth of CCA cells. Previous studies have associated Sphk2 inhibition with both caspase-dependent and -independent cell death, with autophagy being involved in the latter. We have shown that ABC294640 dose-dependently induces apoptosis and this is at least in part through a caspase-dependent pathway. Further, we found that ABC294640 inhibits STAT3 phosphorylation, which is part of a key signaling pathway regulating proliferation and survival in CCA cells. S1P generated from Sphk1 has been shown to promote STAT3 phosphorylation either through its receptor or through inducing IL-6, the main activator of STAT3 [36]. Our study suggests that STAT3 may also be a downstream target of S1P that is generated from Sphk2, which may mediate its pro-survival effect in cancer. Thus, suppression of the STAT3 signaling pathway may be a key mechanism by which Sphk2 inhibition mediates its anticancer activity.

Additionally, we showed that ABC294640 induces autophagy in CCA cells. Although autophagy has been shown to mediate caspase-independent cell death, its role in cancer is still in debate. Emerging evidence suggests that autophagy can promote cancer cell survival by maintaining energy production and is a critical mechanism of therapeutic resistance [23]. Previous reports have shown that ABC294640 promotes autophagy in several cancer types, leading to non-apoptotic cell death. However, whether autophagy plays the same role in CCA is unknown. Therefore we further investigated the effects of autophagy induced by Sphk2 inhibition by ABC294640. Interestingly, we found that inhibition of autophagy using two different inhibitors of autophagy, bafilomycin A1 and chloroquine potentiates ABC294640-induced cytotoxicity and apoptosis. Thus we show for the first time that ABC294640 is capable of inducing a protective autophagy in cancer. Previous studies have shown that CCA cells have high expression of autophagy genes and proteins [37, 38]. Further, inhibition of active autophagy has been shown to inhibit growth and enhance the sensitivity of CCA cells to chemotherapy [37, 39]. We also show that inhibition of autophagy enhances the sensitivity of CCA cells to ABC294640 and promotes its anticancer effect. Our result indicates that there is a cross talk between autophagy and apoptosis in CCA cells. The autophagy inhibitors chloroquine or hydroxychloroquine used either alone or in combination with chemotherapeutics or targeted agents are undergoing evaluation in phase I/II clinical trials across a range of tumor types [23]. Therefore, autophagy inhibition in combination with ABC294640 may be an effective new treatment strategy for CCA.

Inhibition of Sphk2 has been shown to synergize with chemotherapeutic agents in breast cancer cell lines [12]. However, our attempts at combining conventional chemotherapeutic agents such as cisplatin and gemcitabine, the standard chemotherapy for advanced cholangiocarcinoma, were disappointing, with no significant synergy seen (data not shown). We therefore examined the effect of the targeted agent sorafenib, which has been reported to show some efficacy in cholangiocarcinoma, and found that the combination of ABC294640 with this agent was superior to the effect of each agent alone on the WITT and HuCCT1 cell lines. This synergism occur be via more efficient inhibition of STAT3-mediated survival signals.

In conclusion, we show that inhibition of Sphk2 by a novel highly specific inhibitor ABC294640 inhibits proliferation and induces apoptosis in cholangiocarcinoma cell lines. The *in vitro* antitumor activity of ABC294640 correlated with the inhibition of STAT3 signaling. Our data provide preliminary insight into the possible use of ABC294640 as an anticancer drug and Sphk2 as a potential therapeutic target for cholangiocarcinoma treatment. Additionally, our results suggest that combinations of Sphk2 inhibition with sorafenib and/or autophagy inhibitors may provide novel and promising strategies to improve the treatment of cholangiocarcinoma.

## MATERIALS AND METHODS

ABC294640 was synthesized and provided by Apogee Biotechnology Corporation (Hummelstown, PA). Bafilomycin A1 (Baf), chloroquine (CQ), 4,6-diamidino-2-phenylindole dihydrochloride (DAPI), anti-Beclin-1 anti-β-actin primary antibody were purchased from Sigma Aldrich (St. Louis, MO). Sorafenib was purchased from LC Laboratories (Woburn, MA). Pan-caspase inhibitor Z-VAD-FMK was obtained from Merck Millipore (Darmstadt, Germany). Antibodies against human caspase 3 (#9662), caspase 8 (#9746), caspase 9 (#9502), PARP (#9542), LC3B (#2775), p-STAT3 (#9145) and STAT3 (#4904) were purchased from Cell Signaling (Beverly, MA). HyGLO HRP detection kit was from Denville (Metuchen, NJ). Alexa Fluor^®^ 488-labeled goat anti-rabbit IgG was from Life Technologies (Grand Island, NY). The bromodeoxyuridine (BrdU) ELISA kit was from Roche (Indianapolis, IN). High Capacity cDNA Reverse Transcription Kit and Power SYBR^®^ Green RT-PCR Reagents Kit were from Applied Biosystems (Warrington, UK). RNeasy Plus Mini kit was from QIAGEN (Hilden, Germany). Annexin V-FITC Apoptosis Detection kit was obtained from eBioscience (San Diego, CA). All cell culture reagents were obtained from Gibco (Grand Island, NY). ABC294640, sorafenib and Baf were dissolved in dimethyl sulfoxide to make stock solutions of 40 mM, 40 mM and 20 μM respectively. CQ was dissolved in PBS to make a stock solution of 100 mM.

### Cell culture

Six human CCA cell lines HuCCT1, OZ, HuH28, EGI-1, WITT and LIV27 and one normal human cholangiocyte cell line H69 were used. HuCCT1, OZ and HuH28 were obtained from the JCRB. EGI-1 was obtained from DSMZ. WITT was provided by Dr. Tushar Patel (Mayo Clinic, Florida, USA). LIV27 is a novel patient-derived intrahepatic cholangiocarcinoma cell line recently established in our laboratory (Manuscript is in preparation for publication). H69 was provided by Dr. Gregory Gores (Mayo Clinic, Minnesota, USA) and cultured as previously described [40]. HuCCT1, EGI-1 and HuH28 were cultured in RPMI 1640 with 10% FBS. OZ was cultured in Williams's medium with 10% FBS. WITT was cultured in DMEM containing 10% FBS. LIV27 was cultured in DMEM/F12 supplemented with 5% platelet lysate, 0.2% heparin, 1 μg/ml insulin and 0.393 μg/ml dexamethasone. All cells were maintained at 37°C in the presence of 5% CO_2_.

### BrdU cell proliferation ELISA assay

CCA cells were plated in 96-well plates at 3000–10000 cells/well in triplicate according to the characteristics of each cell line. After 24 h, drugs were added and cells were incubated for the indicated time. The BrdU ELISA assay was performed according to the manufacturer's instructions. Cell proliferation was expressed as a percent of control. For analysis of synergy between ABC294640 and sorafenib, cells were exposed to different fixed-ratio combinations of ABC294640 (dose range, 10–50 μM) and sorafenib (dose range, 1.5–7.5 μM) and cell proliferation was assessed by the BrdU ELISA assay after 72 h. The combination index (CI) of ABC294640 and sorafenib was determined using CompuSyn software, based on the median-effect model of Chou-Talalay [41]. A CI < 1 indicates synergism.

### Clonogenic assay

Cells were plated at 500 cells/well in a 6-well plate. After 24 h, drugs were added and cells were incubated for 7 days. Cells were then fixed with methanol-acetic acid (3:1) solution and stained with 0.5% crystal violet. The number of colonies, defined as ≥ 50 cells/colony, was counted manually by light microscopy.

### DAPI staining

Apoptosis was quantified by assessing characteristic nuclear changes of apoptosis after staining with DAPI using fluorescence microscopy as previously described [42]. Apoptosis was defined as the presence of nuclear condensation and fragmentation.

### Annexin V-FITC apoptosis assay

Cells were seeded in 6-well plates at 2 × 10^5^ cells/well and treated with varying concentrations of ABC294640 for the indicated time. Apoptosis was assessed using the Annexin V-FITC Apoptosis Detection kit and performed according to the manufacturer's instructions. Data were analyzed using FlowJo software.

### Immunofluorescence

Cells were cultured and treated with either vehicle or ABC294640 for 24 h. After treatment, cells were fixed with methanol/acetone for 20 minutes. Cells were then washed with PBS and incubated with 0.1% Triton-X-100 for 2 minutes, followed by washing and 1 h blocking in 5% goat serum. Cells were then incubated with antibody against LC3B (1:100) for 1 h at room temperature. Then cells were washed and incubated with secondary antibody (1:200) for 1 h at room temperature, washed again and mounted using Prolong Gold Antifade Mountant with DAPI. Cells were examined by confocal microscopy (LSM 780, Carl Zeiss, Germany). Puncta from 100 to 200 cells were counted from 3 independent experiments for quantitative analysis. Cells displaying more than 5 brightly fluorescent LC3 puncta were counted as positive.

### Western immunoblotting

Equivalent amounts of protein were separated on a 4–15% Tris-HCl gel and transferred to PVDF membranes. Membranes were probed with the appropriate primary antibodies. Blots were then incubated with horseradish peroxidase-conjugated secondary antibodies and signals were visualized using the HyGLO HRP detection kit. β-actin was used as a loading control. Quantitation of the signal was performed using Image J software.

### Real-time qPCR

mRNA was extracted from cultured cells using the RNeasy Plus Mini kit and cDNA was synthesized from 1 μg mRNA using High Capacity cDNA Reverse Transcription Kits according to the manufacturer's instructions. Quantitative real-time PCR was done with the 7300 Real-time PCR System (Applied Biosystems) using the Power SYBR^®^ Green RT-PCR Reagents Kit. 18S was used as the internal control. The primers for Sphk2 were 5′-TTCTATTGGTCAATCCCTTTGG-3′ and 5′-AGCCCGTTCAGCACCTCA-3′. The primers for quantifying 18S were 5′-TTGGAGGGCAAGTCTGGTG -3′ and 5′-CCGCTCCCAAGATCCAACTA-3′.

### Electron microscopy

Cells were fixed with 2.5% glutaraldehyde with 0.1 M sodium cacodylate, washed twice with PBS, fixed further with 1% Osmic acid, dehydrated with a graded ethanol series, embedded, and sectioned. Samples were then embedded and ultrathin (50–60 nm) sections were cut using an ultramicrotome (LKB-I). After staining with 3% uranylacetate and lead citrate, cells were examined with a transmission electron microscope (JEOL USA Inc., Peabody, MA).

### siRNA transfection

HuCCT1 cells were grown in 6-well plates and transfected with 50 nM siRNA using Lipofectamine RNAiMAX (Life Technologies) according to the manufacturer's protocol. The siRNA SphK2 (5′-AACCUCAUCCAGACAGAACGA-3′) and siRNA control duplexes were purchased from RiboBio (RiboBio Co. Ltd., Guangzhou, China).

### Statistics

All data are expressed as the mean ± SEM from at least three independent experiments. All statistical tests were conducted with GraphPad Prism 5.0. The IC_50_ was calculated using nonlinear regression analysis in Prism 5.0. The Student's *t* test was used to compare two groups. In experiments involving more than two groups, one-way ANOVA with a Bonferroni *post hoc* test was used. Results were considered statistically significant at *P* < 0.05.

## SUPPLEMENTARY MATERIALS FIGURES


