# Supplementary material for: Antitumor effect of the novel sphingosine kinase 2 inhibitor ABC294640 is enhanced by inhibition of autophagy and by sorafenib in human cholangiocarcinoma cells
Source: Oncotarget. 2016 Mar 4;7(15):20080–92. doi: 10.18632/oncotarget.7914 (PMC4991440; doi:10.18632/oncotarget.7914)
Supplement: Supplementary file 1 [file oncotarget-07-20080-s001.pdf]

Antitumor effect of the novel sphingosine kinase 2 inhibitor ABC294640 is enhanced by inhibition of autophagy and by sorafenib in human cholangiocarcinoma cells

Supplementary Materials

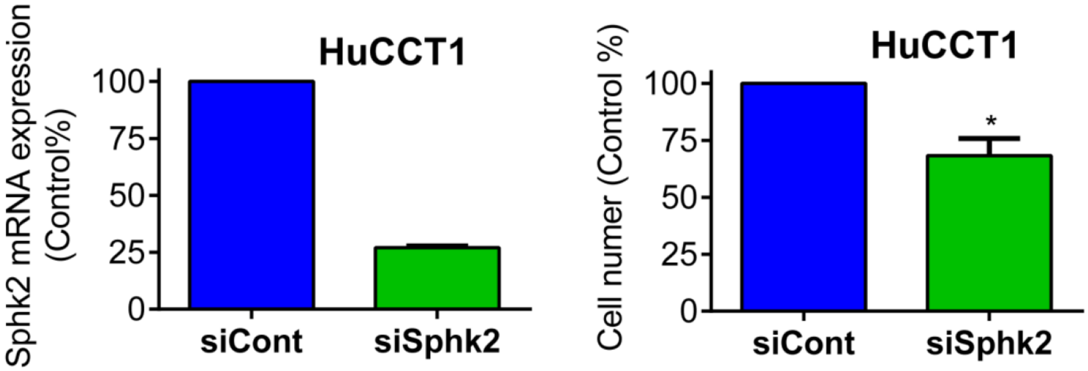

Supplementary Figure S1: Knock down of Sphk2 using specific siRNA inhibits cell proliferation in HuCCT1 cells. HuCCT1 cells were seeded in 6-wells and transfected with siSphk2 (50 nM) or siControl for 72 h. Then Sphk2 mRNA was determined by real-time q-PCR and cell proliferation was determined by cell number counting. Data are mean ± SEM. *$P < 0.05$.
